# Thymic B Cell-Mediated Attack of Thymic Stroma Precedes Type 1 Diabetes Development

**DOI:** 10.3389/fimmu.2018.01281

**Published:** 2018-06-07

**Authors:** Ana Isabel Pinto, Jennifer Smith, Miriam R. Kissack, Karen G. Hogg, E. Allison Green

**Affiliations:** ^1^Centre for Immunology and Infection, Department of Biology, Hull York Medical School, University of York, York, United Kingdom; ^2^Cambridge Institute for Medical Research, University of Cambridge, Addenbrooke’s Hospital, Cambridge, United Kingdom

**Keywords:** type 1 diabetes, thymic B cells, autoreactive antibody, non-obese diabetic, medullary thymic epithelial cells

## Abstract

Type 1 diabetes (T1D) results from a coordinated autoimmune attack of insulin producing beta cells in the pancreas by the innate and adaptive immune systems, beta cell death being predominantly T cell-mediated. In addition to T cells, peripheral B cells are important in T1D progression. The thymus of mice and man also contains B cells, and lately they have been linked to central tolerance of T cells. The role of thymic B cells in T1D is undefined. Here, we show there are abnormalities in the thymic B cell compartment before beta cell destruction and T1D manifestation. Using non-obese diabetic (NOD) mice, we document that preceding T1D development, there is significant accumulation of thymic B cells-partly through *in situ* development- and the putative formation of ectopic germinal centers. In addition, in NOD mice we quantify thymic plasma cells and observe *in situ* binding of immunoglobulins to undefined antigens on a proportion of medullary thymic epithelial cells (mTECs). By contrast, no ectopic germinal centers or pronounced intrathymic autoantibodies are detectable in animals not genetically predisposed to developing T1D. Binding of autoantibodies to thymic stroma correlates with apoptosis of mTECs, including insulin-expressing cells. By contrast, apoptosis of mTECs was decreased by 50% in B cell-deficient NOD mice suggesting intrathymic autoantibodies may selectively target certain mTECs for destruction. Furthermore, we observe that these thymic B cell-associated events correlated with an increased prevalence of premature thymic emigration of T cells. Together, our data suggest that the thymus may be a principal autoimmune target in T1D and contributes to disease progression.

## Introduction

The thymus is a primary lymphoid organ involved in shaping the T cell repertoire. Sequential compartmentalization of developing T cells into the cortical region of the thymus, and subsequently the medulla enable the effective positive and negative selection events, respectively, that are integral in generating an immature T cell repertoire enriched to respond to pathogens but not self-tissue—termed central tolerance (1). Central to this role for the thymus are the medullary thymic epithelial cells (mTECs) (2, 3); capable of autoimmune regulator-driven expression of peripheral tissue-specific antigens (TSAs) (4) and presentation in the context of MHC class I or class II molecules, they trigger events that lead to apoptosis of developing T cells bearing high affinity receptors for self-peptides.

Although extensive studies have documented the importance of mTECs for negative selection of autoreactive T cells (5), other antigen-presenting cell (APC) populations within the thymus have also been shown to participate in T cell-negative selection, particularly dendritic cells (6). A newer member of this family of APCs involved in negative selection is the thymic B cells (7, 8), although it is still not clear how significant their role is in negative selection with respect to that of mTECs and thymic DCs (9). Thymic B cells are present both in man and mice; constituting a minor population of the thymic cellular pool, they are detectable in fetal through to adult mammalians (10, 11). Thymic B cells have a similar phenotype to peripheral B2 cells (12, 13), and their thymic frequency is stable from birth onward. Interestingly, expansion of thymic B cells in myasthenia gravis and systemic lupus erythematosus (SLE) patients (14, 15), or animal models of SLE have been linked to disease progression, suggesting thymic B cells may have a potential role in breakdown of central tolerance (16).

Type 1 diabetes (T1D) is an autoimmune condition where insulin-secreting β cells in the islets of Langerhans are destroyed through coordinated attack by both the innate and adaptive immune systems; the final assault being perpetuated by CD8^+^ cytotoxic T cells (17–19). Defects in central tolerance are linked to emergence of a β cell-specific T cell repertoire (20), yet definitive understanding of the mechanisms underlying defective central tolerance is unclear. Much of our understanding of the immunological events leading to β cell pathology has been derived from the non-obese diabetic (NOD) mouse, a murine model that spontaneously develops T1D with many similarities to those seen in man (21). Studies in NOD mice show T1D is a progressive condition, with priming of the T cell repertoire to β cell antigens in early life followed by infiltration of islets with immune cells (termed insulitis), a period of regulation of the autoreactive response, but ultimately an aggressive and sustained attack on the β cells. It is not clear what immunological event triggers this final stage of the disease.

B cells, too, are known to be important in the T1D process both in man and in NOD mice; abnormally high numbers of islet-infiltrating B cells are linked to rapid progression to T1D in young children (22), and increasing diversity of serum antibodies for β cell antigens increases substantially the risk factor of developing T1D genetically predisposed children (23). In NOD mice, genetic or immunological ablation of B cells protects against T1D development (24, 25), and in both diabetic NOD mice and diabetic patients, depletion of B cells can resolve the condition albeit transiently (26, 27). To date, the role for B cells in T1D progression has been linked to their peripheral APC function—their ability to present β cell antigens to β-reactive CD4^+^ T cells (28) enhances CD4^+^ T helper cell activation of CD8^+^ T cells, and in islets B cells provide survival signals for activated CD8^+^ T cells enabling a sustained cytotoxic attack on β cells (29).

Here, we provide evidence that the thymus of diabetes-prone NOD mice displays evidence of autoreactivity before T1D development. We show that the post-insulitic/prediabetic phase is characterized by abnormally high thymic B cell development, B cell accumulation in follicles at the cortical–medullary junction and the emergence of ectopic germinal centers. Intrathymic autoantibodies bind to undefined antigens on selective mTECs. Subsequently increased mTECs apoptosis, including insulin-expressing mTECs occurs. These events correlate with increased levels of peripheral T cells that have an RAG-GFP phenotype akin to thymocytes that have yet to undergo negative selection, suggesting in NOD mice thymic B cells may contribute to decreased efficacy of negative selection of autoreactive T cells.

Our data provide new insights into thymic abnormalities that precede β cell destruction and highlight the importance of focusing research on these unique thymic B cells as mediators of this chronic condition.

## Materials and Methods

### Mice

C57BL/6 (B6), FVB.RAGp2-GFP reporter mice (30), and NOD.μMT^−/−^ mice (25) have been described elsewhere. FVB.RAGp2-GFP reporter mice were backcrossed 20 generations to either NOD mice (NOD.RAGp2-GFP) or NOD.μMT^−/−^ mice (NOD.μMT^−/−^.RAGp2-GFP mice). All mice used in this study were maintained under specific-pathogen free conditions with a 12 h light–dark cycle and fed normal chow. All animal experimental procedures were carried out in accordance with the Animals and Scientific Procedures Act 1986 were approved by the University of York Animal Welfare and Ethics Review Board and conducted under UK Home Office License approval conforming to ARRIVE guidelines (https://www.nc3rs.org.uk/arrive-guidelines). Diabetes development was determined by assessing urine glucose levels using Diastix (Bayer, Inc.). All animals used in this study were not diabetic. In addition, only female mice were used.

### Antibodies and Flow Cytometry

All antibodies, unless otherwise stated, were purchased from eBioscience. Single-cell suspensions were incubated with antibodies against CD16/32 unconjugated (93), CD3 FITC (145-2C11), CD3 BV421 (17A2; BioLegend), CD4 eFluor450 (RM4-5), CD4 PE (RM4-5), CD4 BV650 (GK1.5; BioLegend), CD8α FITC (53-6.7), CD8β PE-Cy7 (H35-17.2), CD19 eFluor450 (6D5), CD19 PE (6D5), CD19 BV421 (1D3; BioLegend), CD21/CD35 PE (4E3), CD23 PE-Cy7 (B3B4), IgM APC (II/41), IgD eFluor450 (11-26c), IgE BV650 (R35-72; BD Biosciences), IgG PerCP-eFluor 710 (Polyclonal), IgA PE (11-44-2), biotinylated insulin (IBT systems), CD45 PerCPCy5 (30-F11), CD45 BV510 (30-F11; BioLegend); PD1 (29F.1A12; BioLegend), ICOS APC (C398.4A), CD138 BV650 (281-2; BioLegend), CD11b FITC (M1/70), CD11c PE (N418), B220 eFluor450 (RA3-6B2), BCL-6 PerCP-eFluor 710 (BCL-DWN), CXCR5 PE (SPRCL5), IL-21 PE (mhalx21), and Ki67 PE (B5; BD Biosciences). Intracellular labeling of Ki67 was performed using eBioscience kit following manufacturer’s guidelines (catalog number 00-5523-00). Cells were acquired using a BD LSR Fortessa X-20 (BD Biosciences) and data analyzed using FlowJo software^®^ (Tree Star). Doublets were excluded using forward light-scatter gating (FSC-A versus FSC-W) followed by gating on cells based on FSC-SSC. Dead cells were excluded by gating on LIVE/DEAD^®^ Fixable Dead Cell Staining (Thermo Fisher) negative cells. The gating strategies are described in this article in the main figures and supplementary figures, explicit in the axis or described in detail in figures legends. The gates were defined using fluorescence minus one and isotype controls: Rat IgG2a eF450 (eBR2a), Rat IgG2a FITC (eBR2a), Rat IgG2a PE (eBR2a), Rat IgG2a PE-Cy7 (eBR2a), Rat IgG2a APC (eBR2a), Rat IgG2a BV421 (RTK2758, BioLegend), Rat IgG2a BV650 (RTK2758, BioLegend), Rat IgG2a PerCP-eFluor 710 (eBR2a), Rat IgG2b PE (10H5), Armenian Hamster IgG APC (eBio299Arm), Rat IgG1 Biotin (eBRG1) and Rat IgG1 BV650 (RTK2071; BioLegend).

### Detection of Thymic B Cells Bearing Insulin-Specific Receptors

The detection of B cells with receptors that bind insulin has been previously described (31). Briefly, single-cell suspensions isolated from the thymus were incubated overnight at 4°C in PBS supplemented with 1% fetal bovine serum, 1% anti-CD16/32 antibodies (eBiosciences) and biotinylated insulin (0.1 μg/10^6^ cells, ibt systems). Bound insulin was detected with fluorochrome-labeled streptavidin Alexa 6470 (Invitrogen) for 30 min at 4°C. The cells were subsequently incubated with anti-CD19 PE (6D5; eBiosciences), B220 eFluor450 (RA3-6B2; eBiosciences), -CD4 BV650 (GK1.5; BioLegend), CD8β PE-Cy7 (H35-17.2; eBiosciences), -CD45 PerCPCy5.5 (30-F11; eBiosciences) antibodies and LIVE/DEAD Fixable Dead Cell Stains (Thermo Fisher Scientific) for 30 min at 4°C, following which the cells were analyzed by flow cytometry. B cell gates were defined following exclusion of dead cells and T cells (dump channel). All samples were stained with insulin-biotin followed by streptavidin or with streptavidin only, frequencies of B cells insulin^+^ were calculated subtracting the background calculated in sample-matched streptavidin only control.

### Soluble Tissue Extracts and Enzyme-Linked Immunosorbent Assay (ELISA)

Cell-free supernatants from thymic and splenic tissue were prepared as described (32). Briefly, single-cell suspension were centrifuged at 300 *g* for 10 min, 4°C then 15 min, 4°C at 3,000 *g*. Cell-free supernatants were collected and stored at −20°C until analysis. IL-2 and IL-21 cytokines were detected using mouse IL-2 ELISA Ready-SET-Go and mouse IL-21 ELISA Ready-SET-Go ELISA kits following manufacturer guidelines (eBioscience). Isotype classification of immunoglobulins (Igs) in thymic cell-free supernatants or serum was achieved using a rapid ELISA Mouse mAb isotyping kit (Thermo Fisher) following manufacturer’s guidelines.

### Cultures

Bone marrow-derived dendritic cells (BM-DCs) were prepared from the appropriate mice by standard methodology. Immature DCs were pulsed for 16 h with whole insulin (Sigma; 5 µg/ml), LPS (Sigma; 10 ng/ml), or proinsulin peptide pB15-23 peptide (Thermo Fisher; p4878-1; 5 µg/ml). Thymocytes were prepared from mice described in the results and 1 × 10^6^ thymocytes were cocultured in complete RPMI media [10% FCS, 50 µmol/l β-mercaptoethanol, l-glutamine, 50 U/ml penicillin and streptomycin (Life-Sciences)] with 3 × 10^4^ BM-DCs only, or 3 × 10^4^ BM-DCs pulsed with insulin or 3 × 10^4^ BM-DCs pulsed with B15-23 peptide, or anti-CD3 (5 µg/ml) (eBioscience) and anti-CD28 (2.5 µg/ml) antibodies (eBioscience). The cocultures were incubated at 37°C, 5% CO_2_ for 72 h, following which cell proliferation was assessed by flow cytometry. The stimulation index was calculated dividing the frequency of T cells in active proliferation (Ki67^+^) in cells following antigen stimulation by the frequency of T cells in active proliferation in paired non-stimulated culture (background).

For the detection of IL-21, single-cell suspensions from the appropriate tissues were prepared placed in RPMI media (as above) supplemented with 50 ng/ml PMA and 1 µg/ml ionomycin for a total of 5 h at 37°C, 5% CO_2_. Brefeldin A (SIGMA) was added to the cultures at a concentration of 0.4 mg/ml 1 h after the initiation of the culture.

### Immunofluorescence Analysis

Thymi frozen in OCT compound were sectioned (~8 μm) on a cryostat. Sections were fixed in 4% paraformaldehyde or ice-cold acetone then blocked in PBS supplemented with 0.5% BSA. The sections were incubated with unconjugated primary antibodies rabbit anti-mouse IgG (Abcam), rabbit anti-mouse insulin (Abcam), or rabbit anti-mouse cytokeratin V (Abcam) overnight at 4°C. Detection of bound antibody was achieved with goat anti-rabbit IgG Alexa 647 or goat anti-rabbit Ig-Alexa 488 (Invitrogen) or goat anti-rat IgG Alexa 488 (Invitrogen). Anti-B220 directly conjugated with Alexa 647 was incubated for 45 min at room temperature. For detection of apoptosis, following incubation with the secondary antibody an *in situ* apoptosis kit was used (Click-iT™ Plus TUNEL Assay, Alexa Fluor™ 647 dye; Thermo Fisher) according with the manufacturer instructions. Sections were counterstained with DAPI (Molecular Probes) and mounted in Prolong Gold anti-fade or Prolong Diamond (Invitrogen). Confocal microscopy was undertaken using Zen software on a Zeiss LSM 710 fitted on an Axioimager using a 63× (1.4) Plan-Apochromat or 20× (0.6) Neofluor. Binding of autoreactive Ig and TUNEL in microscopy images was quantified using StrataQuest V64 software. Individual nuclei were counted, and the data were presented as scatterplots of mean fluorescence intensity of DAPI versus mean fluorescence intensity of Ig or TUNEL positive cells.

### RNA Isolation and Real-Time RT-PCR Analysis

Thymic tissues were stored at −80°C in RLT. Samples were allowed to thaw, and RNA was carried out using the RNeasy mini kits (Qiagen, Manchester, UK), according to the manufacturer’s instructions. On-column DNase digestion was carried out to remove any contaminating genomic DNA using the RNAse-free DNase set (Qiagen, Manchester, UK) according to the manufacturer’s instructions. The cDNA syntheses were performed with the Superscript II reverse transcriptase system (Invitrogen), according to the manufacture’s instructions. The qRT-PCR of aicda mRNA expression [activation-induced cytidine deaminase (AID) gene] in total thymus was performed with the Taqman qPCR Kit (Applied Biosystems, Warrington, UK). mRNA expression levels were normalized to HPRT1 housekeeping gene using ΔΔCt calculations. Mean relative mRNA expression levels between control and experimental groups were calculated using the 2^−ΔΔCt^ calculations.

### Statistical Analysis

Statistical analyses were performed by parametric or non-parametric tests, selected based on the distribution of the raw data. The comparisons between experimental groups were performed using Student’s unpaired *t*-test, Mann–Whitney, and one-way ANOVA as appropriate. The statistical analyses for fold changes were performed using Wilcoxon signed-rank test. All analyses were conducted using GraphPad InStat (version 5) software (GraphPad).

## Results

### T1D Progression Correlates With Increased Intrathymic B Cell Numbers in NOD Mice

Thymic B cells normally constitute a small population of cells within the murine and human thymus in normal individuals. Abnormality in thymic B cell numbers has been linked to certain autoimmune conditions (14, 15). To determine whether thymic B cell populations differ between diabetes-prone or non-prone mice, we performed time-course flow cytometric studies of age-matched, sex-match NOD and control C57BL/6 (B6) mice.

**Figure 1:**
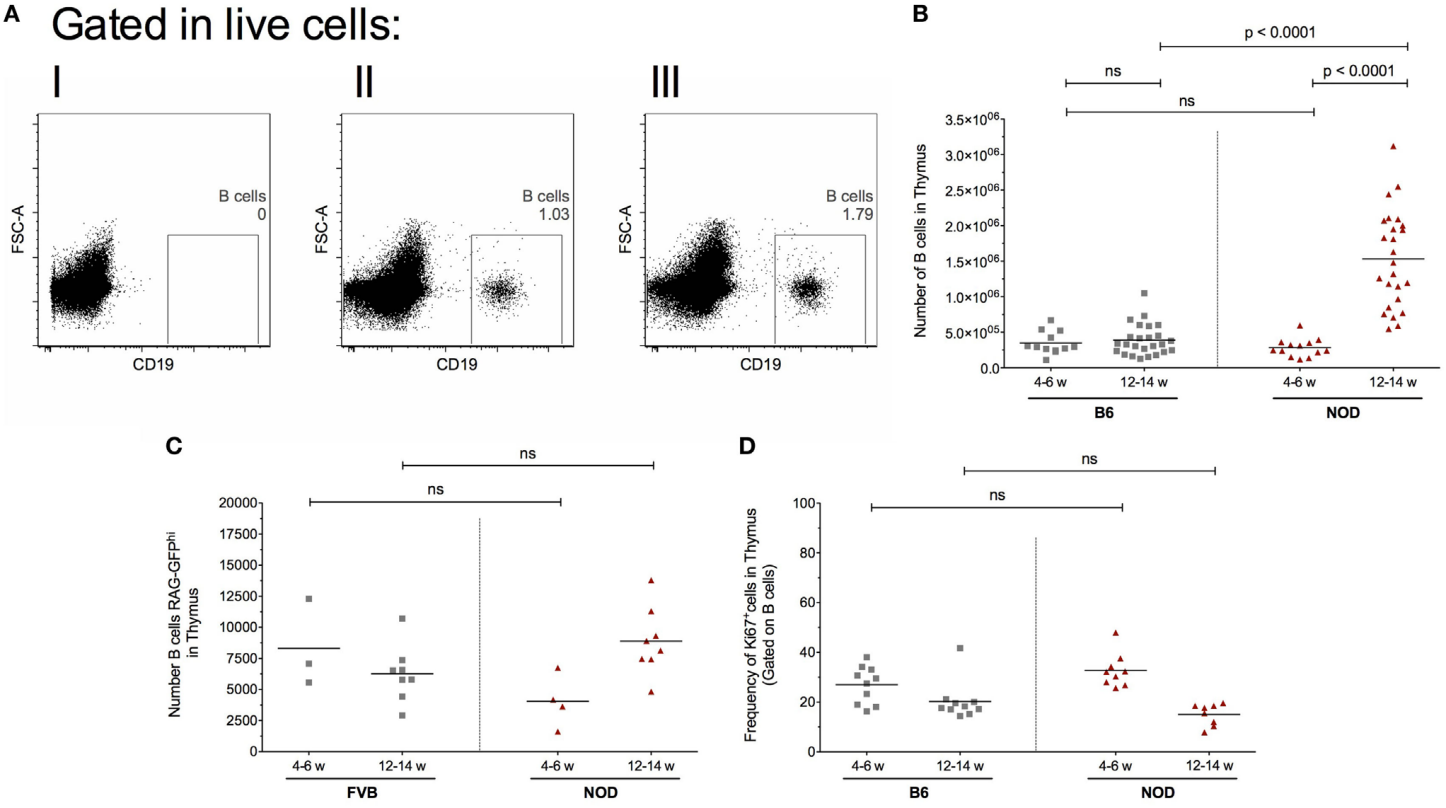
Intrathymic B cell accumulation precedes β cell destruction. Single-cell suspensions were prepared from the respective thymi and all data analyzed on a single cell, live gate. **(A)** Representative dot plots of thymic CD19^+^ cells: (I) isotype control for CD19 antibody; (II) 12-week-old female B6 mouse; and (III) 12-week-old female non-obese diabetic (NOD) mouse. **(B)** Number of B cells in the thymus of 4- to 6-week-old female B6 mice (*n* = 11), 12- to 14-week-old female B6 mice (*n* = 25), 4- to 6-week-old female NOD mice (*n* = 13), and 12- to 14-week-old female NOD mice (*n* = 25). **(C)** Number of RAG-GFP^hi^ B cells in the thymus of 4- to 6-week-old female FVB-RAG-GFP mice (*n* = 3), 12- to 14-week-old female FVB-RAG-GFP mice (*n* = 8), 4- to 6-week-old female NOD-RAG-GFP mice (*n* = 4), and 12- to 14-week-old female NOD (*n* = 8). **(D)** Frequency of Ki67^+^ B cells in the thymus of 4- to 6-week-old female B6 mice (*n* = 10), 12- to 14-week-old female B6 mice (*n* = 10), 4- to 6-week-old female NOD (*n* = 9), and 12- to 14-week-old female NOD mice (*n* = 8). Data presented as scatter plot, each dot equating to a mouse, the bar representing the mean value. Statistical significance determined using the non-parametric Mann–Whitney *U*-test, significant *P* values are shown; ns, not significant.

Diabetes incidence in our female NOD mouse colony is 95%, approximately 3% of mice develop T1D at 12–14 weeks of age, 85% at 18–20 weeks of age with the remaining 7% of females progressing to T1D by 23 weeks of age. Animals not diabetic by 23 weeks of age rarely develop T1D. The data are based on a cohort of 200 animals (Figure S1A in Supplementary Material). This diabetes incidence, combined with the insulitis score—that is the degree of immune cell infiltration of islets and degree of β cell destruction—as mice age (Figure S1B in Supplementary Material) highlight that 12–14 weeks of age in our colony represents late insulitic–preultimate diabetic stage, a critical time when immunoregulation of the autoreactive response starts to breakdown. Thus, in our initial studies, we focused on two major time points; the pre-early insulitic phase (4–6 weeks) and the post-insulitic/prediabetic phase (12–14 weeks) to assess the presence of CD19^+^ thymic B cells. Representative flow cytometry plots for the respective mice are shown in Figure 1A. Although absolute numbers of CD19^+^ B cells remained relatively static in the thymi of control B6 mice at the time points investigated (Figure 1B), with perhaps a slight increase at 12–14 weeks of age, the absolute numbers of CD19^+^ B cells increased significantly in the later age group of NOD mice in comparison with numbers seen either at 4- to 6-week-old NOD mice or 12- to 14-week-old B6 mice. Importantly, the number of thymic B cells at 4–6 weeks of age was comparable between NOD and control B6 mice.

This increased number of thymic B cells in 12- to 14-week-old NOD mice was not related to increased B cell development in the bone marrow, as frequencies of CD19^+^ B cells in this primary lymphoid tissue was comparable between the two strains of mice at both time points investigated (data not shown). These data show that inappropriate accumulation of thymic B cells precedes the overt β cell destruction phase of T1D.

### Intrathymic Signals Trigger Enhanced B Cell Development in NOD Mice

Although previous studies have documented the ability of the thymic environment to enable B cell development in non-autoimmune-prone mice, other reports suggest thymic B cells accumulate *via* periphery B cell migration to the thymus (16, 33). To determine whether the NOD mouse thymus promotes B cell development, we used recombination activation gene green fluorescent protein (RAG2p-GFP) reporter mice on a non-T1D-prone FVB background (hereafter called FVB-RAG-GFP), or on the NOD background (hereafter called NOD-RAG-GFP). In RAG2p-GFP reporter mice, highest GFP expression occurs when RAG genes are active (30). Once recombination of the B cell receptors and T cell receptors is complete and RAG activity is silenced, GFP expression decreases over a 54 h period (30). As such, newly developed B cells can be identified from thymic resident/recirculatory B cells based on the expression of GFP.

Since our control RAG2p-GFP transgenic mice are on an FVB background, we compared thymic B cell frequencies and numbers of this alternative control murine strain to control B6 mice or NOD mice. Although frequencies and absolute numbers of thymic B cells in the FVB strain were higher than the B6 strain, the NOD strain demonstrated significantly greater thymic B cell frequencies and numbers to the FVB strain (Figures S2A,B in Supplementary Material).

We performed time-course, flow cytometry studies of the two strains of mice at the ages shown in Figure 1C, and quantified the number of GFP^hi^ B cells. Representative flow cytometry plots showing the gating strategy for CD19^+^GFP^hi^ B cells is shown in Figure S1C in Supplementary Material. Recently developed CD19^+^GFP^hi^ B cells were readily detectable in both strains of mice at all time points analyzed (Figure 1C). In control FVB-GFP mice, there was no significant changes in B cell development as mice aged. In the NOD strain, although there was no significant change in B cell development when the two age groups were compared, it was clear that thymic B cell development is enhanced as mice enter the late insulitic–prediabetic phase of the T1D pathway.

In light of evidence that the late insulitic–prediabetic phase is characterized by increased B cell development, we asked if homeostatic proliferation of thymic B cells is also affected as mice enter the late insulitic–prediabetic phase. We performed comparative flow cytometric studies between NOD and control B6 mice, assessing for Ki67 expression as a marker for homeostatic proliferation. Interestingly, for both strains of mice, the highest level of homeostatic proliferation of thymic B cells is an early event, with CD19^+^Ki67^+^ B cell frequencies higher in younger mice when compared with older mice (Figure 1D). Furthermore, this decrease in homeostatic proliferation in the 12- to 14-week-old group was more pronounced in NOD mice, although the decrease was not significant.

### The NOD Thymic Environment Has Ectopic Germinal Center Formation Potentiality

To further investigate the phenotype of thymic B cells in NOD mice, we assessed their surface markers. B cells undergo a series of transitions from the immature stage developing follicular (FO) or marginal-zone (MZ) properties. Thus, we qualified the phenotype of thymic B cells assessing for FO (IgM^lo^IgD^+^CD21/35^+^, CD23^+^) versus marginal zone (IgM^+^IgD^lo^CD23^−^CD21/35^+^). We focused our studies on 11- to 14-week-old mice due to the evidence that at this age B cell development is enhanced as are thymic B cell numbers in NOD mice when compared with control mice. Representative flow cytometric plots for our gating strategies are shown in Figure S2D in Supplementary Material.

**Figure 2:**
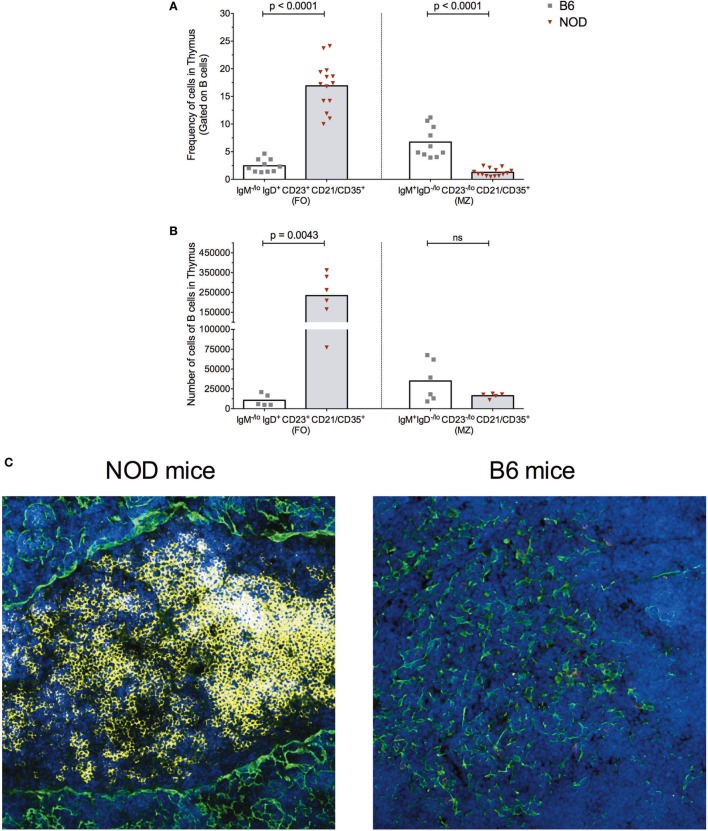
B cells form follicle-like structures at the cortical-medullary junction in non-obese diabetic (NOD) thymi. Flow cytometric analysis of the **(A)** frequency of B cells displaying a follicular (FO) or marginal-zone (MZ) phenotype in the thymus of B6 (*n* = 10) or NOD mice (*n* = 13) and **(B)** absolute number of B cells displaying FO or MZ phenotypes in the thymus of B6 (*n* = 5) or NOD mice (*n* = 6). Comparisons made between aged-matched, female 11- to 14-week-old mice in a single cell, live gate. Data acquired from at least two independent experiments and are presented as scatter plot; *P* values were calculated using the Mann–Whitney *U*-test analysis; ns, not significant. **(C)** Representative confocal immunofluorescence microscopy images of thymi sections examined for B220 (yellow), cytokeratin V (green) expression, and the DNA-intercalating dye DAPI identified nuclei (blue) from 11-week-old female NOD or B6 mice. A total of 14 sections from eight NOD mice and a total of six sections from three B6 mice were analyzed, and there was consistency in the data obtained from the appropriate strains of mice. Confocal fluorescent images were obtained with a Plan-Apochromat 20× objective.

As shown in Figure 2, the frequency of FO B cells within the thymic B cell pool was significantly higher in NOD mice compared with control B6 mice (Figure 2A). This enhancement in FO B cells in the NOD mouse thymus was recapitulated when absolute number of FO B cells was calculated (Figure 2B). By contrast, although the frequency of B cells with an MZ phenotype was significantly decreased in the NOD mouse thymus compared with control B6 mouse thymus, the absolute numbers of these cells were similar between the two strains of mice.

The increased numbers of FO B cells in NOD mice with respect to B6 control mice led us to investigate whether the thymic B cell form follicle-like structures. Immunohistochemical studies revealed B cell follicle-like structures form only in NOD mice (Figure 2C). Initially, B cells are detectable at the cortical–medullary junction at 9 weeks of age in NOD mice (data not shown) with pronounced accumulation of B cells into follicle-like structures in this location by 11 weeks of age. The presence and location of B cell follicle-like structures was identical irrespective of whether we used anti-B220 or anti-CD19 antibodies to identify B cells (data not shown) confirming the accumulating B220^+^ cells are not plasmacytoid DCs. We quantified the number of B cell follicle-like structures in the thymus of 9- to 11-week-old NOD mice; of 15 individual sections assessed, 90% contained one follicle, 5% two follicles, and 5% no follicles.

**Figure 3:**
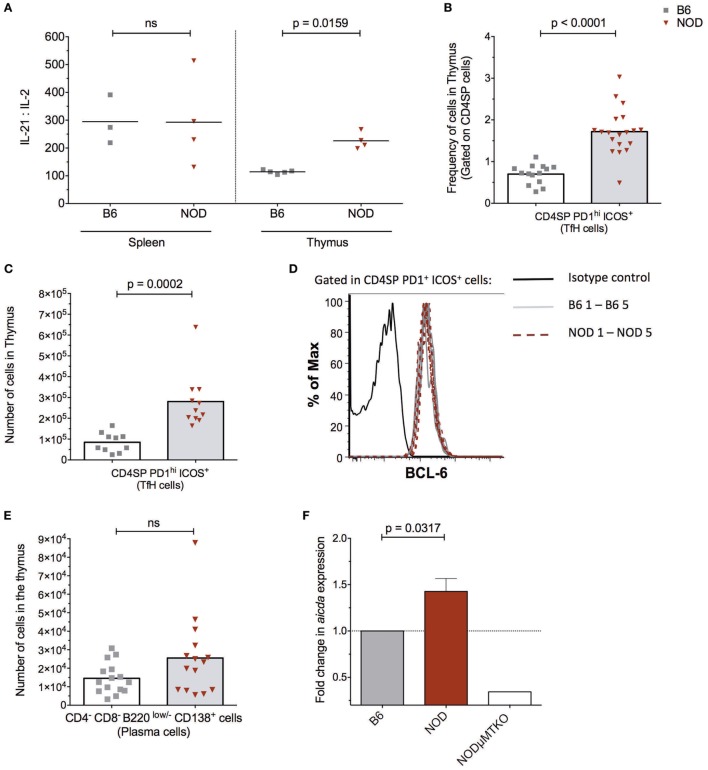
The non-obese diabetic (NOD) thymus has the hallmarks of ectopic GC development. **(A)** Evaluation of IL-2/IL-21 ratio in cell-free supernatants from spleen and thymic tissue from 11- to 15-week-old B6 (*n* = 5) or NOD (*n* = 4) mice. The data shown are representative of two individual experiments showing similar results. **(B)** Frequency of CD4^+^ T follicular helper (TfH) cells in the thymus of B6 (*n* = 12) and NOD mice (*n* = 17). **(C)** Number of CD4^+^ TfH cells in the thymus of B6 (*n* = 10) and NOD mice (*n* = 11). **(D)** Representative histogram of BCL-6 expression in CD8^−^CD4^+^PD1^hi^ICOS^+^ cells in thymus of NOD (*n* = 5) and B6 (*n* = 5) mice. **(E)** Number of plasma cells in the thymus of B6 (*n* = 15) and NOD mice (*n* = 15). For panels **(B–E)**, comparisons made between female, age-matched 10- to 14-week-old B6 and NOD mice. The analysis was performed on a single cell, live gate, and the data are presented as a scatterplot, each dot equating to a mouse, the bar represents mean value. *P* values were calculated using the Mann–Whitney *U*-test analysis and are shown in this figure; ns, not significant. The data are pooled from at least two independent experiments giving similar results. **(F)** Quantitative-PCR analysis of activation-induced cytidine deaminase (AID) mRNA (*aicda* expression) levels in the whole thymus. Data were normalized to HPRT mRNA as described in Section “Materials and Methods,” and fold change in NOD mice AID mRNA when compared with normalized AID mRNA levels for B6 mice. All mice were 11–14 weeks of age, a total of five female B6 mice were compared with five female NOD mice. One thymic sample from a female B cell-deficient NODμMT^−/−^ mouse was used as a negative control. The data are pooled from two independent experiments and are presented mean ± SEM. *P* values were calculated using the Mann–Whitney *U*-test and are shown in this figure; ns, not significant.

The presence of follicle-like structures in the thymus of late insulitic–prediabetic NOD mice, but not control B6 mice, lead us to ask if the thymic environment could support germinal center formation. Of interest was the relationship between IL-2 and IL-21, the latter being a key mediator of germinal center formation; the cytokine promotes B cell somatic hypermutation and class switching, and the development and maintenance of T follicular helper (TfH) cells (34). In NOD mice, IL-21 has been associated with T1D progression (35, 36) and CD4^+^CD45R^−^ T cells isolated from T1D patients secrete greater quantities of IL-21 than quantified from normal individuals (37). We prepared cell-free supernatants (32) from the thymi of NOD and control B6 mice at the ages shown in Figure 3A and performed ELISA assays. As a comparison, we analyzed cell supernatants from the spleens of the same mice. The results were tabulated as ratio of IL-21:IL-2. No differences were seen in IL-21:IL-2 ratios in splenic preparations from the two strains of mice. However, the NOD mouse thymus had a significant bias in IL-21 concentrations in comparison with B6 mice.

In light of this IL-21 bias, we quantified the frequency and absolute numbers of CD4SP cells that expressed a TfH cell phenotype in the thymus of the two strains of mice. As shown in Figures 3B,C, NOD mice exhibited a significant increase in frequencies and absolute numbers of CD4SPPD1^hi^ICOS^+^ T cells, and these cells also expressed transcription factor Bcl-6 (Figure 3D) and CxCR5 (Figure S3A in Supplementary Material). In addition, approximately 5% of NOD putative thymic TfH cells secreted IL-21, a frequency that was comparable to that seen for splenic TfH cells from the same mice (Figure S3B in Supplementary Material). Furthermore, this increase in thymic TfH cells in NOD mice in comparison with B6 control mice correlated with an increased number of CD4^−^CD8^−^B220^low/−^CD138^+^ plasma cells in the NOD mouse thymus, although this increased number was not significant (Figure 3E; Figure S3C in Supplementary Material). Together, these data suggested that ectopic germinal centers could be present in the NOD mouse thymus, but absent in control B6 mouse thymus. To support this hypothesis, we looked for a *bona fide* germinal center marker; the enzyme AID. RNA was prepared from thymi isolated from NOD mice or control B6 mice and quantitative real-time RT-PCR performed. As an additional control, we included thymic mRNA isolated from age-matched, sex-matched NOD-μMT^−/−^ mice. The relative expression of transcripts for AID in NOD mice was normalized to control B6 mice. As shown in Figure 3F, the NOD mouse thymi has enhanced AID expression in comparison with control B6 mice. Thus, ectopic germinal center formation is likely a feature of the NOD thymus and precedes the preultimate β cell destruction phase of T1D.

### Thymic Immunoglobulins Binding Selective mTECs Correlates With mTEC Apoptosis

**Figure 4:**
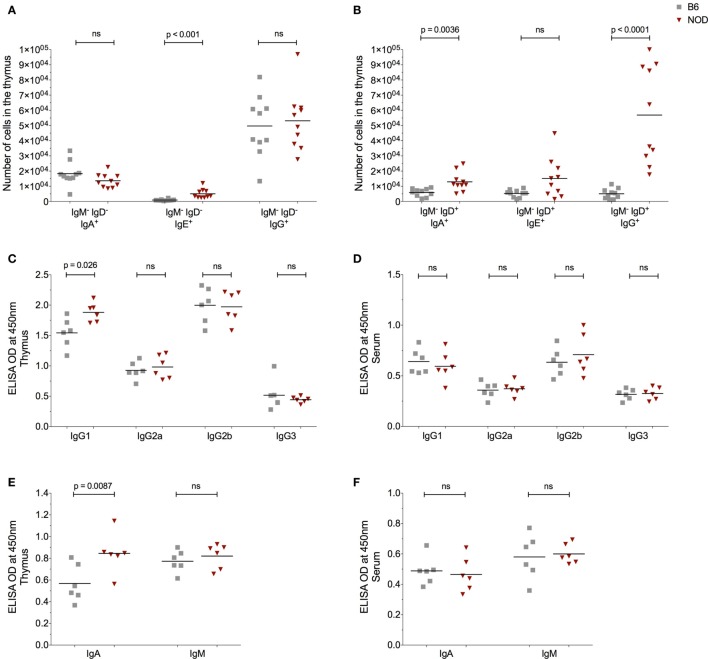
The non-obese diabetic (NOD) thymus harbors a unique pattern of immunoglobulin (Ig) isotypes. **(A)** Number of IgM^−^IgD^−^IgA^+^, IgM^−^IgD^−^IgE^+^, and IgM^−^IgD^−^IgG^+^ B cells in the thymus of 11- to 14-week-old female B6 (*n* = 10) or female NOD mice (*n* = 10). **(B)** Number of IgM^−^IgD^+^IgA^+^, IgM^−^IgD^+^IgE^+^, and IgM^−^IgD^+^IgG^+^ B cells in the thymus of 11- to 14-week-old female B6 (*n* = 10) and female NOD mice (*n* = 10). **(C–F)** Optical density (OD) values of the respective Igs in cell-free tissue supernatants **(C,E)** or serum **(D,F)**. A total of six female B6 and six female NOD mice were assessed in two independent experiments. Data are presented as scatter plot, each dot equating to one mouse and bar representing the mean. *P* values were calculated using the Mann–Whitney *U*-test analysis and are shown in this figure; ns, not significant.

The presence of AID and enhanced plasma cell frequencies in the NOD thymus with respect to control B6 mice, made us query the Ig isotype of the thymic B cells and secreted antibodies. Since we previously had investigated the IgM^+^ B cell thymic subtype (Figure 2), this time we focused on class-switched IgM^−^ cells. The number of IgM^−^IgD^−^IgA^+^ and IgM^−^IgD^−^IgG^+^ B cells was similar in the thymus of both NOD and control B6 mice as determined by flow cytometry (Figure 4A). By contrast, the number of IgM^−^IgD^−^IgE^+^ B cells was significantly increased in the NOD mouse thymus with respect to control mice. Interesting, a unique population of IgM^−^IgD^+^ B cells [similar to those reported in T1D patients (31)] was detectable in the thymic tissue. These IgM^−^IgD^+^ B cells dually expressed IgG, IgE, or IgA with the number of dual expressing IgD^+^IgA^+^ and IgD^+^IgG^+^ B cells being significantly higher in the NOD mouse thymus than the B6 control mouse thymus, the most significant being the IgD^+^IgG^+^ isotype (Figure 4B; Figure S4A in Supplementary Material). By contrast, no differences in IgD^+^IgE^+^ B cell numbers were seen between the two strains of mice. We next assessed the isotype of soluble thymic Ig by ELISA, in comparison with serum Ig. Only the IgG1 and IgA Ig isotypes were enhanced in the thymi of NOD mice in comparison with control B6 mice (Figures 4C,E). By contrast, IgG2a, IgG2b, and IgM antibody levels were similar in both strains of mice, with IgG3 antibody levels being slightly lower in NOD mice than in the thymus of B6 control mice. Interestingly, in NOD mice thymus, B cells predominately used the kappa light chain, there being a significant decrease in the presence of lambda light chains when compared with B6 control mice (Figure S4C in Supplementary Material). In addition, this isotype pattern documented in the NOD mouse thymus seemed unique for this tissue, as similar ELISA-based isotyping of Igs in the serum of the two strains of mice revealed little difference in levels of each isotype assessed (Figures 4D,F). However, similar to the thymus, in the serum, there was a significant decrease in lambda light chain usage in NOD mice in comparison with B6 controls (Figure S4B in Supplementary Material). Quantification of the thymic Ig isotypes supported the data that IgG1 and IgA are significantly greater in the thymus of NOD mice with respect to control B6 mice (Figure S5A in Supplementary Material).

**Figure 5:**
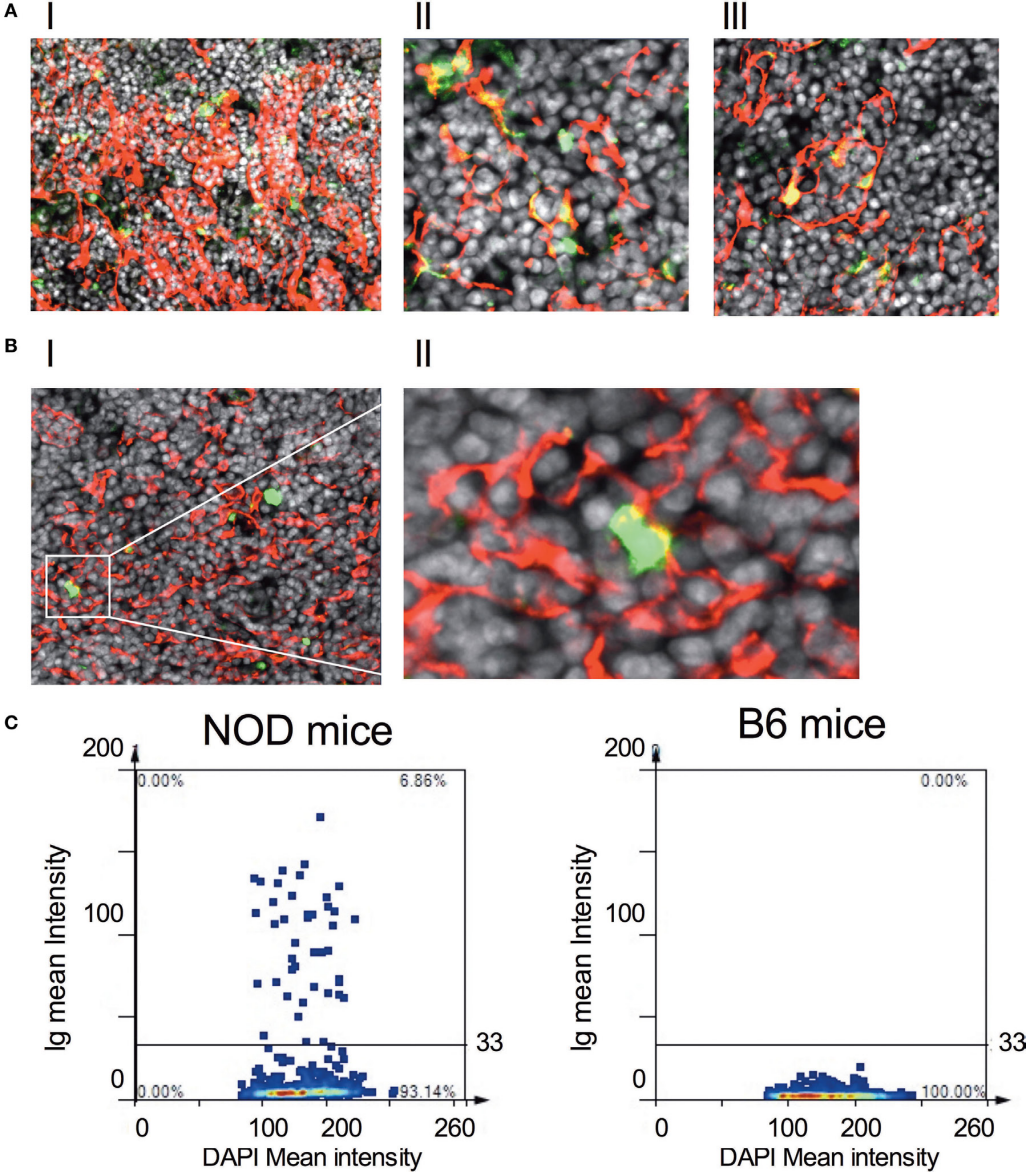
IgGs bind to thymic stromal components in non-obese diabetic (NOD) mice. **(A,B)** Representative confocal immunofluorescence microscopy images of thymi sections of NOD [**(A)**, I–III] and B6 mice [**(B)**, I–II] examined for cytokeratin V (red), murine IgG (green), and the DNA-intercalating dye DAPI (white). A total of six 11-week-old NOD mice and five 11-week-old B6 mice, two sections per mouse were examined. [**(A)**, I–II] is derived from different NOD mice. The confocal fluorescent image in AI was obtained with a Plan-Apochromat 20× objective to give a broader view of the extent of immunoglobulin bound to thymic stroma, arrows indicating some of the cells co-positive for cytokeratin V and mouse IgG. The confocal fluorescent images in AII and AIII were obtained with a Plan-Apochromat 63× objective. For panel **(B)**, the confocal fluorescent image was obtained using a Plan-Apochromat 20× objective. **(C)** Quantification of murine Ig-bound to stromal cells of age-matched 11-week-old, female NOD or B6 mice. Confocal immunofluorescence microscopy images were subjected to StrataQuest V64 analysis, a total of 2–3 × 10^4^ DAPI^+^ cells/mm^2^ were counted, and the mean fluorescence intensity of DAPI^+^ cells versus mean fluorescence intensity of anti-Ig is presented as a scattergram. The data shown are representative of two independent mice examined giving similar results.

We decided to explore further these thymic B cells to determine whether they harbored receptors specific for islet autoantigens, focusing on their specificity for insulin (31). Representative gating strategy for identifying insulin-reactive B cells is shown in Figure S5B in Supplementary Material. Although the frequency of cells bearing receptors specific for insulin is significantly less in the thymus of NOD mice with respect to control B6 mice within the B cell fraction, absolute numbers of insulin-reactive B cells was similar in NOD mice and B6 control mice (Figure S5C in Supplementary Material). Thus, insulin-reactive B cell numbers do not correlate with T1D susceptibility at this time point. Due to this finding, we decided to ask whether thymic B cells produce antibodies that target, as yet, undefined antigens on thymic stroma. Thymic tissue sections from 11-week-old NOD mice were incubated with anti-mouse antibodies that would detect any mouse Ig bound to thymic stroma *in situ* and bound antibodies were detected by confocal microscopy. To qualify whether any Igs that bound to thymic stroma interacted with mTECs, we included mTEC-binding anti-cytokeratin V antibodies in the assay. As shown in Figure 5A, there was detectable binding of murine Igs to thymic stroma in NOD mice, suggesting these cells had murine Igs bound to them *in situ*. Interestingly, intrathymic Igs were bound almost exclusively to cytokeratin V^+^ mTECs, and it appeared that only a proportion of mTECs were being targeted by the Igs. In contrast to NOD mice, there was substantially less intrathymic Ig in control B6 mice interacted with thymic stroma, particularly cytokeratin V^+^ mTECs (Figure 5B). Furthermore, there was no evidence of intrathymic Igs bound to thymic stroma, including cytokeratin V^+^ mTECs in B cell-deficient NOD-μMT^−/−^ mice confirming the specificity of the anti-mouse antibodies for mouse Igs (Figure S6 in Supplementary Material). We quantified the frequency of cells with murine Ig bound in the thymus of 11- to 14-week-old NOD and B6 mice. We selected images that had comparative frequencies of cytokeratin V^+^ mTECs and counted 2–3 × 10^4^ DAPI^+^ cells/mm^2^. As shown in Figure 5C, approximately 7% of cells had bound murine Ig in the NOD mouse thymus. By contrast, the frequency of cells bound by murine Igs in B6 mice was so low as to be undetectable.

**Figure 6:**
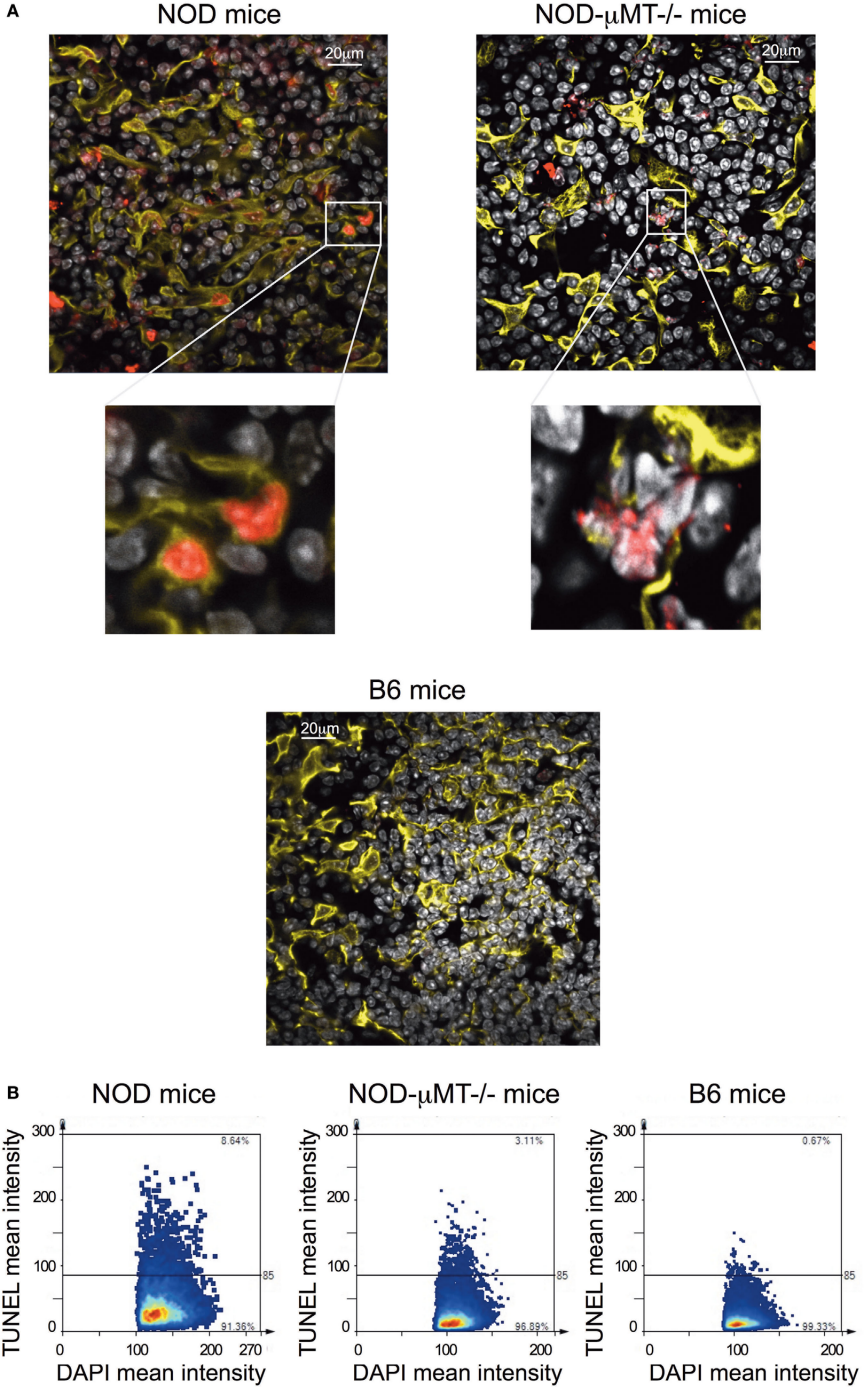
Increase in thymic B cells was associated with increased apoptosis of stromal cells. **(A)** Representative confocal immunofluorescence microscopy images of thymi sections from 9- to 14-week-old female non-obese diabetic (NOD), and NOD-μMT^−/−^ and B6 mice examined for cytokeratin V (yellow), apoptosis (red), and the DNA-intercalating dye DAPI (white) expression. The data are representative of similar data acquired from six female NOD, six female NOD-μMT^−/−^, and four female B6 mice, three sections per mouse were examined. In all cases, the confocal fluorescent images were obtained with a Plan-Apochromat 63× objective. Bar represents 20 µm. **(B)** Quantification of Tunel^+^ stromal cells of age-matched 11-week-old, female NOD, NOD-μMT^−/−^ or B6 mice. Confocal immunofluorescence microscopy images were subjected to StrataQuest V64 analysis, a total of 4 × 10^4^ DAPI^+^ cells/mm^2^ were counted, and the mean fluorescence intensity of DAPI^+^ cells versus mean fluorescence intensity of Tunel is presented as a scattergram.

Finally, we queried the significance of *in situ* binding of thymic stroma by Igs, particularly the potential that a selective number of mTECs underwent apoptosis. In this regard, we incubated thymic tissue sections from 11-week-old, female NOD mice with antibodies specific to cytokeratin V^+^ and assessed for apoptosis by confocal microscopy following Tunel staining (Figure 6A). As controls, we similarly analyzed thymic tissue sections from control B6 mice and NOD-μMT^−/−^ mice. The inclusion of NOD-μMT^−/−^ mice was important to determine whether the diabetes-associated MHC class II molecules unique to NOD mice was sufficient to trigger mTEC apoptosis *via* non-B cell-mediated mechanisms. Thymic tissue sections from NOD mice had clear evidence of apoptosis, and such apoptotic cells were almost exclusively cytokeratin V^+^ mTECs. Apoptosis of cytokeratin V^+^ mTECs was also evident in NOD-μMT^−/−^ mice, although the proportion of apoptotic cells seemed lower than that for B cell sufficient NOD mice. In contrast to the NOD strains, we could not see any apoptotic cells in the B6 control mouse thymic tissue section. We quantified the frequency of apoptotic cells in the thymic sections of the respective strains of mice (Figure 6). We counted a total of 4 × 10^4^ DAPI^+^ cells/mm^2^ per section, ascertaining similar frequencies of cytokeratin V^+^ mTECs for each tissue sections examined. As shown in Figure 6B, ~6% of DAPI^+^ cells were apoptotic in NOD mice. This frequency of apoptosis was twofold higher than seen for NOD-μMT^−/−^ mice (~3%). In contrast to the NOD strains, <1% of cells were apoptotic in control B6 mice. We were curious to determine if apoptotic cytokeratin V+ mTECs in NOD mice expressed insulin. Thymic tissue sections from 11-week-old female NOD mice were incubated with anti-cytokeratin V^+^ and anti-insulin antibodies and apoptosis determined by TUNEL staining as before. As a control, we similarly analyzed thymic tissue sections from age-matched, female B6 mice. Interestingly, within the apoptotic cytokeratin V^+^ mTEC pool in NOD mice resided cytokeratin V^+^ mTECs that expressed insulin, although it is important to note that some insulin^+^ cytokeratin V^+^ mTECs were not apoptotic suggesting there is not a complete loss of insulin^+^ cytokeratin V^+^ mTECs but a reduction in their numbers. Similarly, some apoptotic mTECs did not express insulin (Figure S7 in Supplementary Material).

Taken together, these data suggest that B cell-mediated autoimmune targeting of cytokeratin V^+^ mTECs results in the loss of a distinct population of cytokeratin V^+^ mTECs, some of which express insulin, and this key feature occurs before sustained autoimmune attack in the pancreas.

### Thymic B Cells Enhance Premature Egress of T Cells From the Thymus

**Figure 7:**
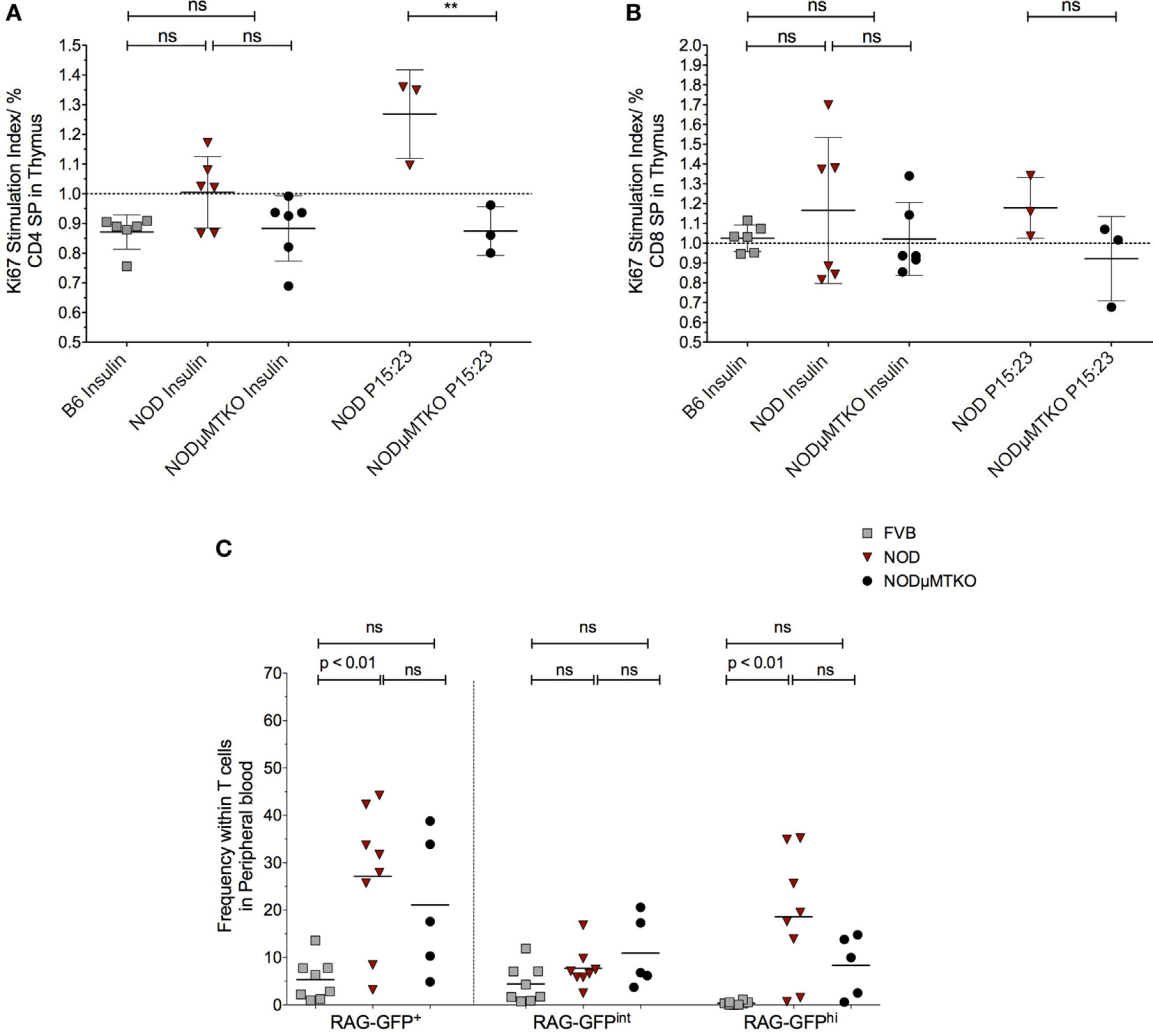
B cells promote premature thymic-release of T cells before negative selection. **(A,B)** Thymocytes from 11- to 12-week-old B6, non-obese diabetic (NOD) and NOD NOD-μMTKO mice were stimulated with insulin or B15:23 peptide (NOD and NOD-μMTKO mice, only) for 72 h and Ki67 expression in CD4SP **(A)** or CD8SP **(B)** cells as a measure of proliferation was determined by flow cytometry. The frequency of Ki67^+^ cells for stimulated samples was normalized against the frequency of Ki67^+^ cells in unstimulated samples. **(C)** Frequency of RTEs (RAG-GFP^hi^) in peripheral blood of 11- to 12-week-old FVB-GFP (*n* = 8), NOD-GFP (*n* = 8), or NOD-μMT^−/–^ GFP (*n* = 5) mice. Data are pooled from two independent experiments, and cells were analyzed on a live, single, CD3^+^ T cell gate. The data are presented as scatter plot, the bar representing the mean value; *P* values were calculated using the two-way ANOVA followed by Tukey multiple comparison test and are shown this figure; ns, not significant.

The evidence that thymic stroma had bound autoantibodies and the presence of these autoantibodies correlated with increased apoptosis of thymic stroma, including some insulin^+^ mTECs, we investigated the impact this may have on thymocytes capable of responding to islet antigen, particularly insulin. We isolated the thymocytes from NOD mice thymi and cultured the cells in the presence of BM-DCs and either whole insulin or proinsulin peptide 15:23 (38). The proliferative response to the CD4SP and CD8SP thymocytes to the respective stimulants was assessed by flow cytometric analysis of Ki67 (Figure 7; Figure S8A in Supplementary Material). As controls, we included B6 mice stimulated with whole insulin, and B cell-deficient NOD-μMT^−/−^ mice stimulated with whole insulin or proinsulin peptide 15:23. For CD4SP cells only those isolated from B cell sufficient NOD mice responded to whole insulin, although the response was not significant in comparison with control mice (Figure 7A). The responses to whole insulin for thymocytes from B6 and NOD-μMT^−/−^ mice being close to baseline. By contrast, CD4SP thymocytes from NOD mice exhibited a significantly increased response to proinsulin P15:23 with respect to NOD-μMT^−/−^ mice. The responses of CD8SP thymocytes were slightly different; whereas thymocytes isolated from NOD mice responded to the whole insulin molecule, the responses for individual mice were quite diverse—some responded well, others’ response close to baseline levels for B6 control mice (Figure 7B). Similarly, CD8SP thymocytes from NOD-μMT^−/−^ mice had some diversity in responsiveness to whole insulin, although it was noted that even the best responders still responded weaker than that seen for NOD mice. By contrast, CD8SP thymocytes from NOD mice responded far better to proinsulin P15:23, than those from NOD—μMT^−/−^ mice, although the response was not significantly enhanced. In these same mice, the responses to the proinsulin peptide were less diverse and above baseline levels.

We initially wondered whether this increased response for insulin and proinsulin peptide by NOD thymocytes was representative of increased survival of autoreactive T cells, and thus a breakdown in negative selection. In particularly, we queried whether thymocytes that had very recently rearranged their TcR escaped from the thymus before completing negative selection. If this held true, we expected an increase in RAG-GFP^hi^ T cells in the blood; RAG-GFP levels normally fall during negative selection due to the time to complete the process and as such, peripheral T cells are usually RAG-GFP^int^ (30). To test this hypothesis, we performed flow cytometry analysis of total GFP levels of T cells in the peripheral blood of NOD-RAG-GFP mice in comparison with control FVB-RAG-GFP mice and B cell-deficient NOD-μMT^−/–^ RAG-GFP mice. Representative flow cytometry plots showing the gating strategy is shown in Figure S8B in Supplementary Material. As shown in Figure 7C, in the NOD murine strains, the frequency of total GFP^+^ T cells in peripheral blood was greater than seen for the control FVB strain, for NOD mice this increase being significant. Furthermore, this increased frequency of GFP^+^ T cells in the peripheral blood of the NOD strains was almost entirely due to GFP^hi^ cells, as GFP^int^ cells were only slightly increased in frequency in comparison with control FVB-RAG-GFP mice, again NOD mice showing a significant increase. Importantly, although not significant, it was clear that the frequency of RAG-GFP^hi^ cells in B cell sufficient NOD-RAG-GFP mice was higher than in B cell-deficient NOD-μMT^−/–^ RAG-GFP highlighting the importance of B cells in the early release of T cells from the thymus before negative selection.

## Discussion

Ablation of efficient purging of autoreactive T cells in the thymus and the role of B cells in T1D seem two distinct entities in understanding how immunological tolerance is broken in this chronic autoimmune condition. Here, we establish that inappropriate accumulation of B cells in the NOD mouse thymus is a unique feature of the disease process, and these thymic B cells may play a role in the egress of pre-negatively selected T cells.

Type 1 diabetes progression in both man and NOD mice occurs over time. The initial stages of T1D, where priming of the immune response to islet antigen occurs but not overt β cell destruction, is characterized by autoantibodies to β antigens (39). It is accepted that following priming of the autoreactive T cell repertoire to β cell antigens, the activity of the autoreactive T cells is kept in check by regulatory mechanisms. Ultimately, such regulation fails, and leading to β cell destruction. Little is known as to why regulation of autoreactive T cells fails over time, although paucity of, or dysfunction of, T regulatory cells is speculated to contribute to the phenomenon (40–42). Our data add a new dimension to our understanding of the immunological changes that occur at the late insulitic– prediabetic phase that may tip the autoreactive T cell response in favor of β cell destruction; targeted thymic B cell autoimmune attack of thymic stroma expressing β cell antigens.

B cells are present in the thymus of mammals from fetal age to adulthood, their numbers remaining relatively static in ontogeny and equating to those of thymic dendritic cells (5, 11, 13). Previous studies in NOD mouse strains documented B cell accumulation in the thymus of aged mice (43, 44). Here, we extended on these early studies showing that in NOD mice, thymic B cell numbers are not static, their numbers significantly increase at the late insulitic–prediabetic phase suggesting the restricted B cell niche normally present expands. This change in B cell numbers occurs at the same time as increased numbers of RAG^+^ B cells are detected in the thymus, but decreased homeostatic proliferation. Together these findings suggest that permissiveness of B cell development that can normally occur within the thymus (33, 45–47) is enhanced in NOD mice as they age, and the increase in B cell numbers potentially reflects this increased rate of development rather than *in situ* proliferation. Although we cannot exclusively discount that peripheral B cells migrating to the thymus contribute to the thymic B cell population, we, like others, have found peripheral B cells have little propensity to traffic to the thymus [data not shown (47)]. Future studies in how the NOD mouse thymic environment potentially nurtures B cell development and retention will be informative.

The phenotype of thymic B cells in NOD mice resembles that of thymic B cells in non-autoimmune strains of mice; they predominantly express B2 FO cell markers, and have a predominantly activated phenotype with high MHC and costimulatory molecule expression [data not shown (45, 48)]. The location of thymic B cells in NOD mice is also reminiscent of reports in other murine strains- positioned predominantly at the cortico-medullary junction- but in contrast to non-autoimmune prone mice, large B cell follicles form and this is age-dependent. Furthermore, the hallmarks of germinal centers are readily detectable in the NOD thymus; IL-21 and TfH cells. Abnormalities in levels of IL-21 and TfH cells in peripheral tissues, and blood, have been strongly associated with T1D (37, 49). Here, we show similar abnormalities exist in the thymus occurring specifically at the late insulitic–prediabetic phase of the T1D condition. In addition, the thymus of NOD mice has enhanced levels of AID mRNA transcripts, suggesting increased *in situ* somatic hypermutation and class switching of the B cell repertoire activity. Plasma cells are also increased in the thymus of NOD mice with respect to control animals which taken all this information together implies ectopic germinal centers are a feature of the NOD thymus, not just their pancreas (50). Our evidence that the NOD mouse thymus is populated with significantly increased numbers of B cells with IgG, IgA, and IgE receptors with respect to non-autoimmune prone mice, as well as enhanced levels of soluble IgG1 and IgA antibodies supports our rationale of ectopic germinal center formation in this primary lymphoid tissue.

The significance of these unique changes in the NOD mouse thymus as mice progress along the T1D pathway, we believe, is that they have the potential to impact on the capacity of negative selection of autoreactive T cells to occur effectively. The importance of mTEC expression of TSAs for efficient deletion of developing T cells bearing autoreactive T cell receptors is well established (51). Our evidence that a selective population of mTECs have autoantibodies bound *in situ*, and in the presence of thymic B cells, a proportion of mTECs undergo apoptosis, a number of which express insulin, is likely to have implications on negative selection of islet-reactive T cells. The antigenic specificity of the intrathymic autoantibodies target is unknown, and we do not believe that they must recognize insulin to impact of T1D progression. It is possible that the intrathymic autoantibodies recognize and promote apoptosis of particular mTECs that express certain TSAs that are associated with other autoimmune conditions NOD mice develop (52–54). It follows that reduction in insulin-expressing mTECs may happen inadvertently. Alternatively, or in addition, it is possible that thymic cognate B–T cell interactions promote survival of developing autoreactive T cells as opposed to their deletion (7).

Our data are supportive of the rationale that pre-negatively selected T cells are potentially released from the thymus prematurely. Two-photon microscopy has documented that developing T cells reside in medulla for 3–5 days to complete negative selection (55). In RAG2p-GFP reporter mice, this duration in the medulla equates to decreased GFP intensity due to the 54 h half-life of the molecule (30). Our evidence that in NOD mice CD3^+^GFP^hi^ cells are significantly enhanced in peripheral blood with respective to non-autoimmune prone mice suggests an aborted time, or failed entry into, the medulla of GFP^hi^ T cells, and as a consequence failed negative selection. It follows that the increased export of non-negatively selected T cells could overpower waning regulatory mechanisms in the islets leading to the final sustained attack of the β cells.

The fledgling field of thymic B cell research is starting to unravel the importance of this unique population of cells in the immune system. Our data highlight a new relationship between thymic B cells and T1D development. Future studies that define the *in situ* developmental pathway and receptor specificity of thymic B cells will be important for identifying key therapeutic strategies for T1D and other autoimmune conditions in which thymic B cells make a contribution.

## Ethics Statement

All animal procedures were approved by the University of York Animal Welfare and Ethics Review Board and conducted under UK Home Office Licence approval conforming to ARRIVE guidelines.

## Author Contributions

AP, JS, MK, and EG performed the experiments. AP, JS, MK, KH, and EG analyzed the data. AP and EG wrote the paper.

## Conflict of Interest Statement

The authors declare that the research was conducted in the absence of any commercial or financial relationships that could be construed as a potential conflict of interest.
