# Supplementary material for: Thymic B Cell-Mediated Attack of Thymic Stroma Precedes Type 1 Diabetes Development
Source: Front Immunol. 2018 Jun 7;9:1281. doi: 10.3389/fimmu.2018.01281 (PMC5999731; doi:10.3389/fimmu.2018.01281)
Supplement: Supplementary file 1 [file data_sheet_1.docx]

**Supplementary Material**

**Thymic B cell-mediated attack of thymic stroma precedes Type 1 Diabetes Development.**

Ana Isabel Pinto, Jennifer Smith, Miriam R. Kissack, Karen G. Hogg and E. Allison Green*

*Correspondence: Dr. E. Allison Green; [allison.green@york.ac.uk](mailto:allison.green@york.ac.uk)

**% islets**

(b)

No insulitis

Peri-insulitis

Insulitis

Destructive insulitis


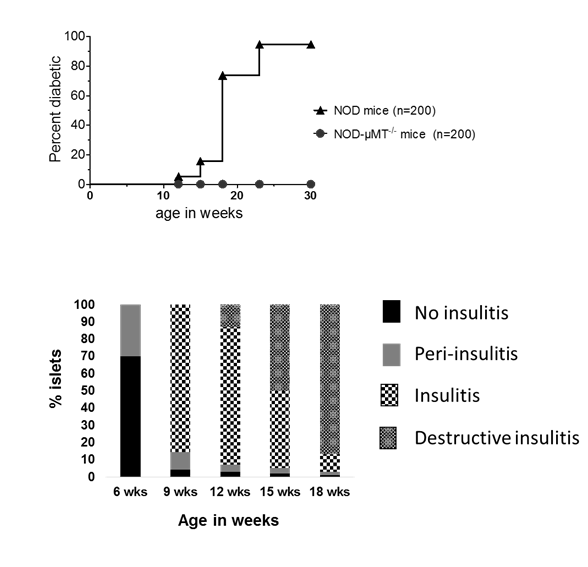


a

b

**Supplementary Figure 1. Diabetes association and insulitis scoring in female NOD mice.** (a) Diabetes development was monitored in female NOD or NOD-μMT^-/-^ mice over a 30 week observation period. The data is presented as a Kaplan-Meier survival curve. (b) Pancreatic frozen tissue sections from female NOD mice at the ages shown (n=5/age group) were incubated with anti-insulin antibodies and DAPI. A total of 60 islets were scored per age group for immune cell infiltration and insulin expression as a marker of viable β cells. Peri-insulitis: <50% of islet infiltrated, intact β cells; insulitis:>50% of the islet infiltrated, <2% β cell destruction and, destructive insulitis: >50% of the islet infiltrated, few intact β cells present.


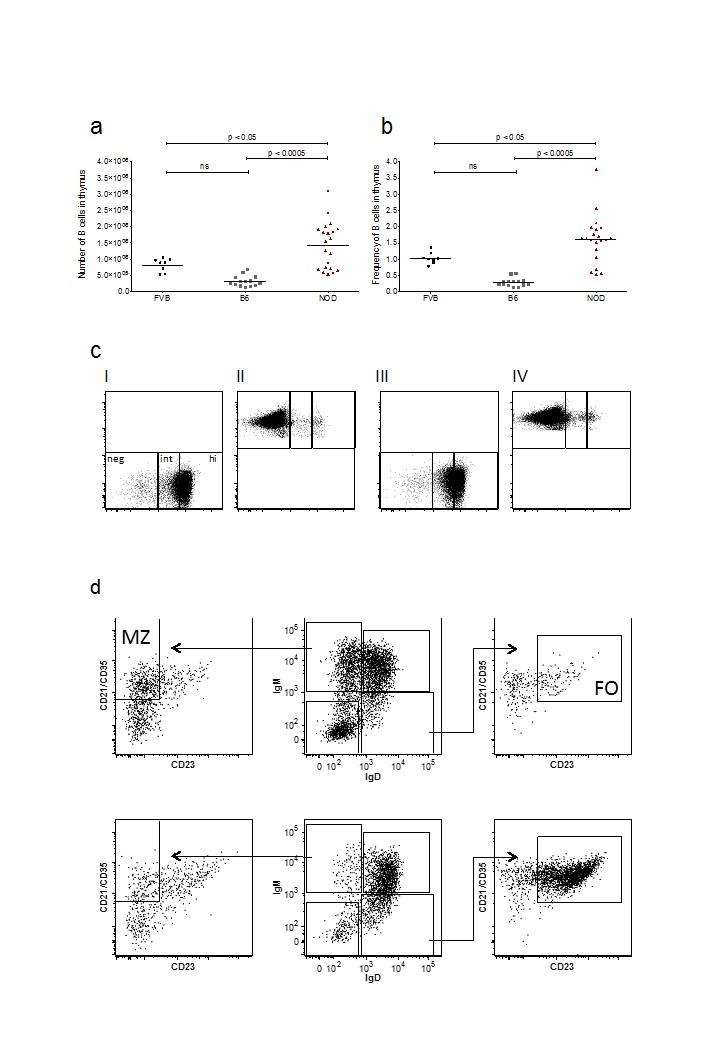


Supplementary Figure 2. Thymic B cell frequencies and absolute numbers are comparable between B6 and FVB mice. Comparisons made between aged-matched, female mice from diabetes non-susceptible strains of mice to that of female NOD mice. (a-b) The frequency (a) and absolute numbers (b) of B cells in the thymus of 11-14 week old FVB-RAG-GFP mice (n=8), B6 mice (n=13), and NOD (n= 21) are shown. The cells were analysed on a live, single cell gate. The data is presented as scatter plot, the bar representing the mean value; P values were calculated using the two-way Anova followed by Tukey multi comparison test are shown in the figure, ns= not significant. (cI-IV) Representative dot plots of RAG-GFP expression of thymocytes from 12 week old FVB-RAG-GFP (I, II) and NOD-RAG-GFP mice (III, IV): (I, III) gated in DP cells; x-axis GFP, Y-axis CD19, (II, IV) gated on CD19^+^ cells; x-axis GFP, Y-axis CD19. (d) Representative dot plots for characterization of Follicular (FO) and Marginal-zone (MZ) B cells based on the expression of IgM, IgD, CD23 and CD21/35 on live, single CD19^+^ cells in the thymus of 11-14 week old B6 mice (upper panels) and NOD mice (lower panels).

**
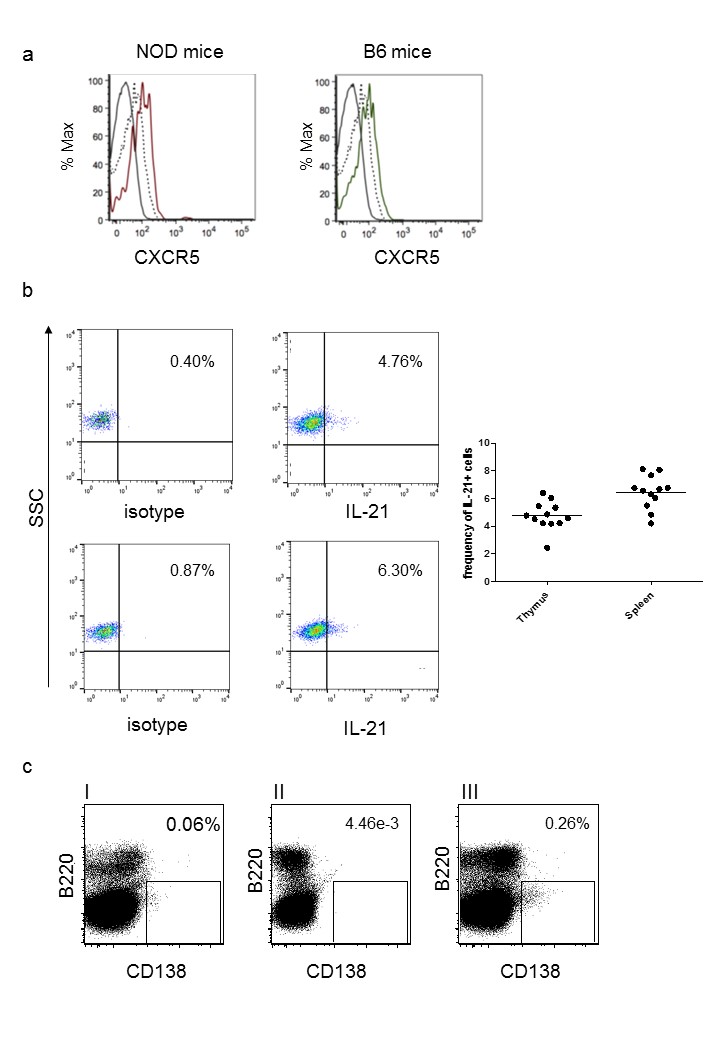
**

**Supplementary Figure 3. Characterizing T follicular helper cells and plasma cells.** (a) Representative histograms of CxCR5 expression (black, green and red lines) on CD4^+^ cells from 11 week old, female NOD or B6 mice. Live, single cells were gated on CD4SPPD^-^1^-^ICOS^+^ (bold black line) or CD4SPPD-1^+^ICOS^+^ (red or green lines) cells. Dotted lines are isotype control gated on CD4SPPD-1^+^ICOS^+^ cells. The data shown is representative of one mouse of five examined for each strain, each mouse giving identical results. (b) Representative flow cytometry plots and scatterplots of IL-21 expression in thymic or splenic Follicular T helper cells from 11 week old, female NOD mice. The cells were gated on live, single cell SPCD4^+^ICOS^+^ cells. A total of 12 mice were examined, each dot of the scatterplot representing one mouse, the bar representing the mean value. (c) Representative dot plots for the gating strategy for assessing presence of B220^lo^ CD138^+^ plasma cells within a live, single cell, CD45^+^ CD4^-^ CD8^-^ thymocyte gate: (I) Isotype control for anti-CD138 antibodies; (II) 12 week old, female B6 mouse; and (III) 12 week old, female NOD mouse.

**Supplementary Figure 4 – Thymic B IgM^-^ IgD^+^ cells are class switched and soluble Lambda Light chains are enhanced in thymus of NOD mice.** (Relates to Fig.4) **(a-b)** Representative dot plots for characterization of B cells based on the expression of IgM, IgD, IgA, IgE and IgG: (aI-aIV) Isotype controls for Igs staining gated in live single CD19^+^ cells in the thymus of 11-14 week old NOD mice; (bI) IgM and IgD expression gated in live single CD19^+^ cells in the thymus of 11-14 week old NOD mice; (bII) IgA expression gated in live single CD19^+^ IgM^-^ IgD^+^ cells in the thymus of 11-14 week old NOD mice; (bIII) IgE expression gated in live single CD19^+^ IgM^-^ IgD^+^ cells in the thymus of 11-14 week old NOD mice; IgG expression gated in live single CD19^+^ IgM^-^ IgD^+^ cells in the thymus of 11-14 week old NOD mice. (c-d) The optical density of light chain usage in cell free tissue supernatants (b) or serum (c) was measured by ELISA. Data is pooled from two independent experiments. Comparisons made between aged-matched, sex-match 11-12 week old B6 (n=6) and NOD (n=6) mice. Data is presented as a scatter plot each dot representing a mouse, the bar represents the mean value. P values were calculated using the Mann-Whitney U-test analysis and are shown in the figure, ns= not significant.


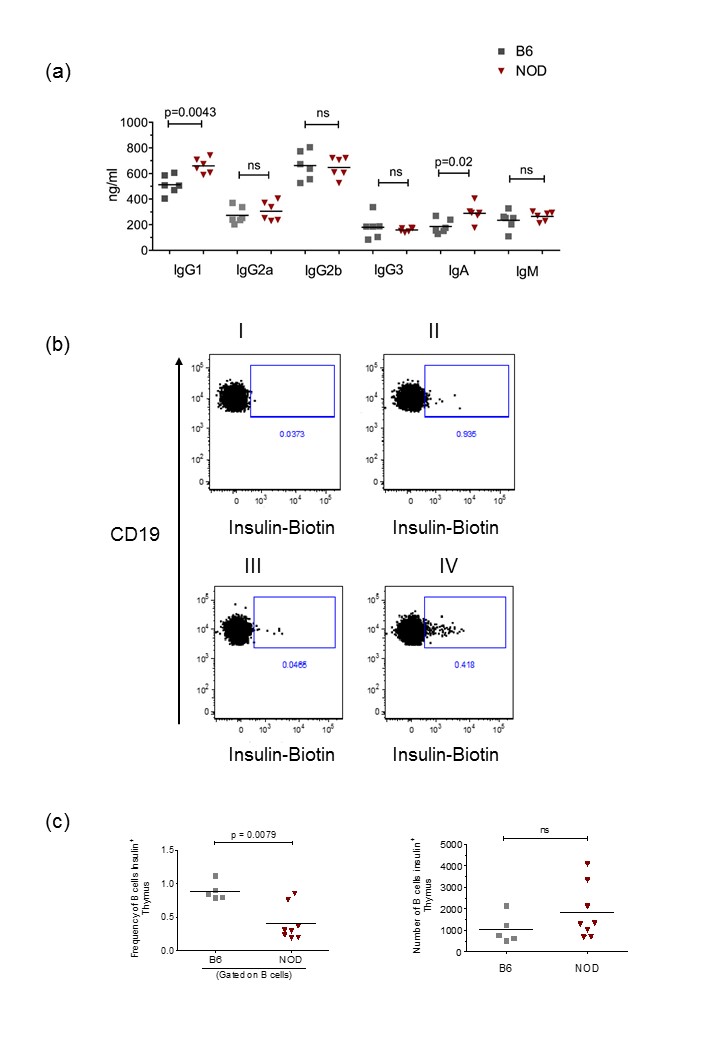


**Supplementary Figure 5. Quantification of thymic Ig concentrations.** (a) The concentrations of Ig isotypes in cell-free supernatants of thymic tissue from age-matched, female NOD or B6 mice was determined by quantitative ELISA. Data is presented as a scatter plot each dot representing a mouse, the bar represents the mean value. P values were calculated using the Mann-Whitney U-test analysis and are shown in the figure, ns= not significant. (b) Representative dot plots for the gating strategy used to assess the frequency of thymic B cells bearing insulin receptors within a live, single cell, CD45^+^ CD4^-^ CD8^-^ CD19^+^ B220^+^ thymocyte gate: (I) Streptavidin only control staining in thymocytes recovered from B6 mouse; (II) frequency of B cells bearing insulin receptor in B6 mouse; (III) Streptavidin only control staining in B cells recovered from NOD mouse; and (IV) frequency of B cells bearing insulin receptor in NOD mouse. (c) Frequency and absolute numbers of B cells expressing receptors for insulin in the thymus of 11-14 week old B6 (n=5) and NOD (n=8) mice. Data is pooled from two independent experiments and analysis performed on a single cell, live CD45^+^ CD4^-^ CD8^-^ CD19^+^ gate. The data is presented as a scatterplot, the bar representing mean value. P values were calculated using the Mann-Whitney U-test analysis and are shown in the figure, ns= not significant.


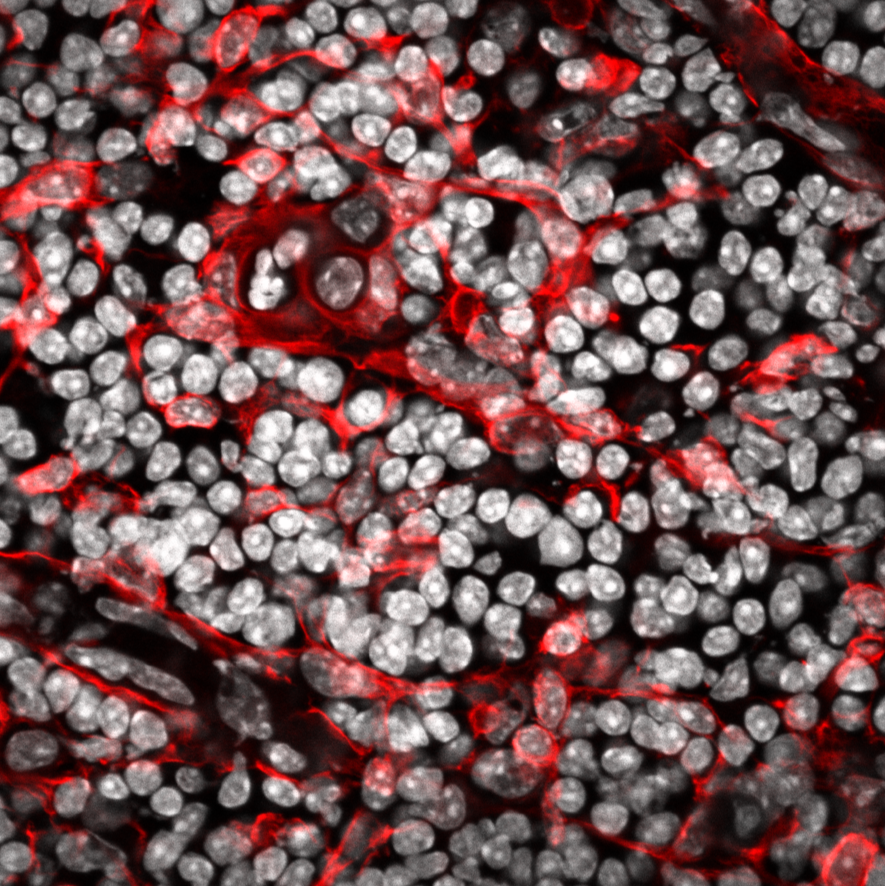


**20μm**

**Supplementary Figure 6. B cell deficient NOD-uMT-/- mice do not have thymic immunoglubulins bound to thymic stroma.** Representative confocal fluorescent image of thymic medulla from NOD-uMT^-/-^ mice showing absence of murine immunoglobulin bound to mTECs *in situ*. 7-8μm sections were incubated with anti-cytokeratin V antibodies (red), anti-murine immunoglobulin antibodies (green) and nuclei visualized with the DNA-intercalating dye DAPI (white). The data was acquired using a Plan-Apochromat 63x objective. The image represents one image from 15 sections examined (three sections per mouse from 5 individual mice examined) all giving identical results.


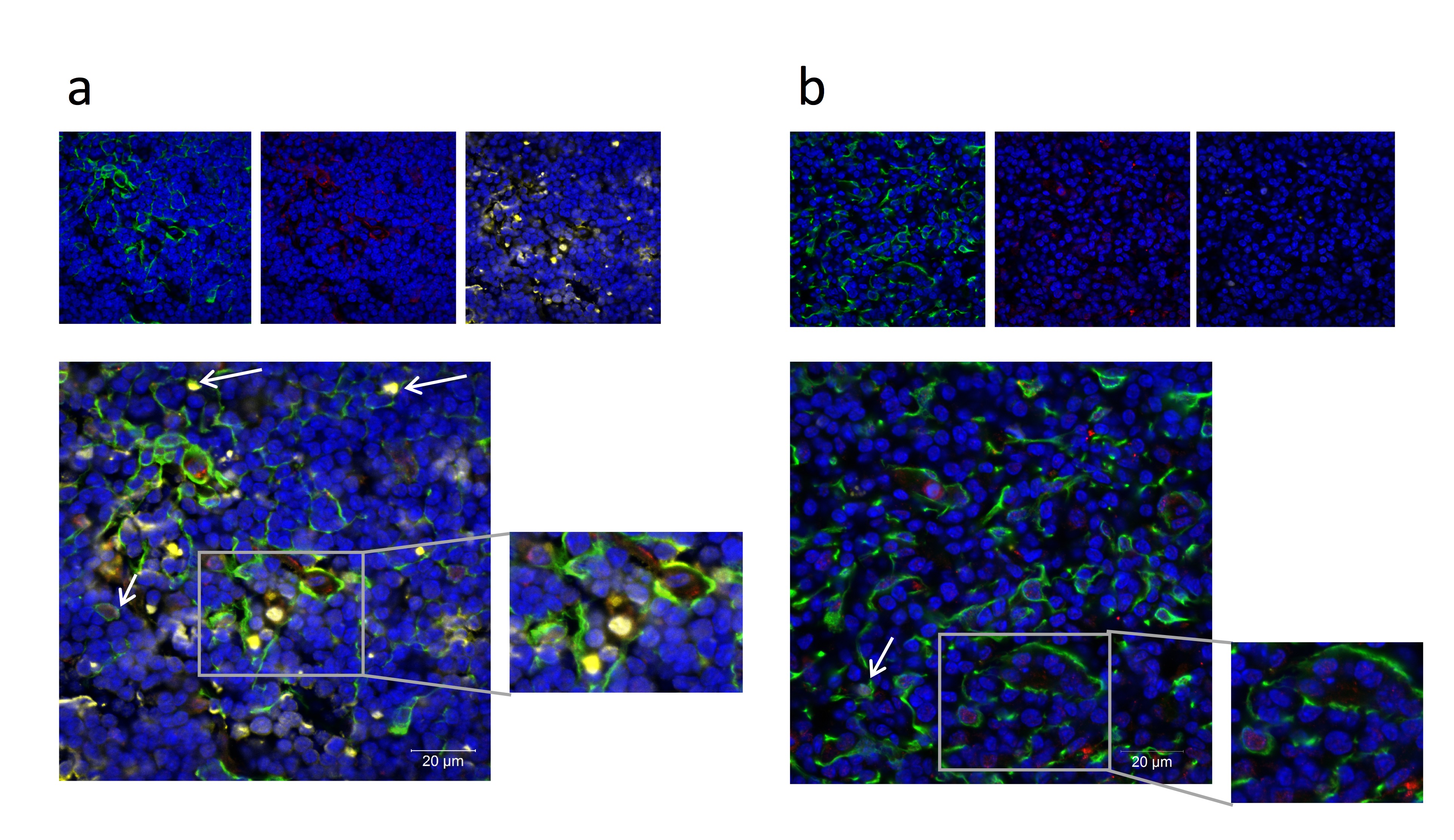


**Supplementary Figure 7 – Increase in thymic B cells was associated to increased apoptosis of stromal cells.** (a and b) Representative confocal immunofluorescence microscopy images of thymi sections from 9-14 week old NOD (a) and B6 mice (b) examined for cytokeratin V (Green), insulin (Red), apoptosis (Yellow), and the DNA-intercalating dye DAPI (blue) expression. The data is representative of 6 NOD and 4 B6 mice, and two sections per mouse. Upper panels in (a) and (b): DAPI and cytokeratin V (left), DAPI and insulin (middle) and DAPI and tunel. Lower enlarged panel: DAPI-cytokeratin V-insulin-tunel merged. Arrows represent additional areas of cytokeratin V-insulin-tunel triple positive cells. In all cases, the confocal fluorescent images were obtained with a Plan-Apochromat 63x objective. Bar represents 20µm.


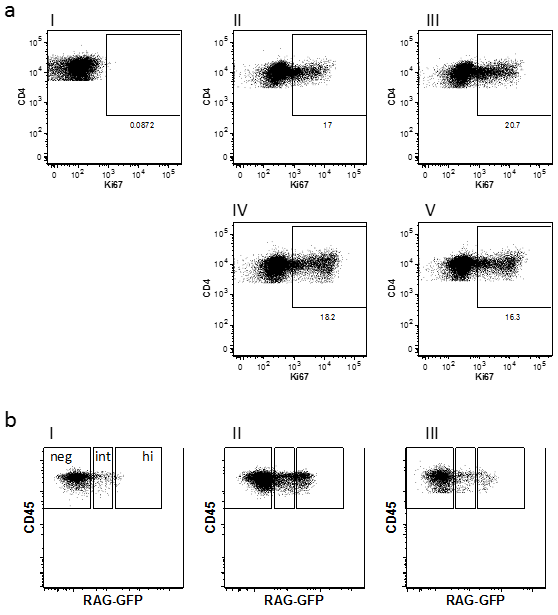


**Supplementary Figure 8. Flow cytometry gating strategy for assessment of proliferating T cells in culture based on Ki67 expression and in characterizing Recent Thymic Emigrant (RTE) populations in circulation based on the expression intensity of RAG-GFP.** (a) All representative dot plots were gated on live, single, CD3^+^ CD4^+^ cells: (I) Isotype control for Ki67, (II) Ki67 expression in unstimulated thymocytes recovered from NOD mouse, (III) Ki67 expression in thymocytes stimulated with the peptide P15:23 recovered from NOD mouse, (IV) ) Ki67 expression in unstimulated thymocytes recovered from NODμMT^-/-^ mouse, (V) Ki67 expression in thymocytes stimulated with the peptide P15:23 recovered from NODμMT^-/-^ mouse. (b) All representative dot plots were gated on live, single, CD3^+^ cells (T cells): (I) FVB-RAG-GFP mouse; (II) NOD-RAG-GFP mouse; and (III) NOD-μMT^-/-^-RAG-GFP mouse. All mice analysed were 11-13 weeks of age.
